# A closed-tube methylation-sensitive high resolution melting assay (MS-HRMA) for the semi-quantitative determination of *CST6* promoter methylation in clinical samples

**DOI:** 10.1186/1471-2407-12-486

**Published:** 2012-10-22

**Authors:** Lampros Dimitrakopoulos, Panagiotis A Vorkas, Vasilis Georgoulias, Evi S Lianidou

**Affiliations:** 1 Laboratory of Analytical Chemistry, Department of Chemistry, University of Athens, Athens, 15771, Greece; 2 Department of Medical Oncology, University General Hospital of Heraklion, PO BOX 1352, Crete, 71110, Greece; 3 Present address: Biomolecular Medicine, Department of Surgery and Cancer, Imperial College London, London, SW7 2AZ, UK

**Keywords:** Methylation-sensitive high-resolution melting analysis, Cystatin M, CST6, DNA methylation, Breast cancer, Methylation specific PCR

## Abstract

**Background:**

*CST6* promoter is highly methylated in cancer, and its detection can provide important prognostic information in breast cancer patients. The aim of our study was to develop a Methylation-Sensitive High Resolution Melting Analysis (MS-HRMA) assay for the investigation of *CST6* promoter methylation.

**Methods:**

We designed primers that amplify both methylated and unmethylated *CST6* sequences after sodium bisulfate (SB) treatment and used spiked control samples of fully methylated to unmethylated SB converted genomic DNA to optimize the assay. We first evaluated the assay by analyzing 36 samples (pilot training group) and further analyzed 80 FFPES from operable breast cancer patients (independent group). MS-HRMA assay results for all 116 samples were compared with Methylation-Specific PCR (MSP) and the results were comparable.

**Results:**

The developed assay is highly specific and sensitive since it can detect the presence of 1% methylated *CST6* sequence and provides additionally a semi-quantitative estimation of *CST6* promoter methylation. *CST6* promoter was methylated in 39/80 (48.75%) of FFPEs with methylation levels being very different among samples. MS-HRMA and MSP gave comparable results when all samples were analyzed by both assays.

**Conclusions:**

The developed MS-HRMA assay for *CST6* promoter methylation is closed tube, highly sensitive, cost-effective, rapid and easy-to-perform. It gives comparable results to MSP in less time, while it offers the advantage of additionally providing an estimation of the level of methylation.

## Background

DNA methylation is one of the most frequent epigenetic events in the mammalian genome that usually occurs in regions rich in CG dinucleotides. Alterations in DNA methylation are very common in cancer cells; many tumor suppressor genes which are normally unmethylated, when they undergo aberrant DNA methylation are silenced and as a consequence they are not expressed [1]. In particular, hypermethylation has been reported as an early event in breast cancer [2], frequently leading to gene silencing through methylation of CpG-rich regions near the transcriptional start sites of genes that regulate important cell functions [3]. DNA methylation is believed to be an early event in the process of cancer development and progression since tumor suppressor genes are frequently inactivated at very early stages in human cancer. Thus, DNA methylation is considered as a promising biomarker for early detection and prognosis estimation in cancer patients [4,5].

Sodium bisulfite (SB) modification of DNA is necessary for DNA methylation assays that are based on PCR amplification, since DNA polymerase does not recognize methylated nucleotides, and as a result methylation information is lost during amplification. Through bisulfite treatment this information is maintained, since unmethylated cytosines are transformed into uracils, while 5-methylcytosines remain unaffected. There are two different approaches, which allow DNA methylation analysis through PCR amplification of SB modified DNA. The first approach is based on design of primers that specifically amplify methylated or unmethylated templates, and is adopted by methylation specific PCR (MSP) and quantitative MSP. The second approach is based on primers that amplify a region of the desired template including CpG islands, no matter what its methylation status is. In this case, Methylation Independent PCR (MIP) is firstly performed and information on the methylation status of that region is obtained through post-PCR analyses techniques like bisulfite sequencing, restriction digestion, single-strand conformation analysis, and high-resolution melting [6].

High-Resolution Melting Analysis (HRMA) firstly introduced in 2003 [7] has several advantages for clinical analysis, since it is a closed-tube, probe-free technique, rapid, simple, cost-effective and non-destructive. Initially developed for mutation scanning and genotyping studies [8-12], high-resolution melting technology can be useful for the detection of methylation as well. Recently, the development of a new generation of melting instrumentation and the introduction of highly sensitive fluorescent dye chemistries, allowed the development of Methylation-Sensitive High-Resolution Melting Analysis (MS-HRMA). MS-HRMA is based on the different melting profiles of unmethylated and methylated PCR products, due to their different sequence composition (CG content) [6]. MS-HRMA is characterized by high sensitivity, reproducibility and accuracy, while it is a closed tube method less prone to contamination problems [13].

Cystatin M or E/M (encoded by the *CST6* gene) is an endogenous inhibitor of lysosomal cysteine proteases that functions to protect cells against uncontrolled proteolysis [14]. Cystatin M was first identified and cloned by Sotiropoulou et al. by differential RNA display as a transcript that was significantly down-regulated in metastatic breast cancer cells when compared to primary breast cancer cells [15]. Later, the same protein was identified and cloned independently from embryonic lung fibroblasts and was named Cystatin E [16]. Cystatin E/M is a low molecular mass protein sharing 27-32% homology with other cystatins. Cystatin M has been assigned to chromosome region 11q13 [17], which is the site of loss of heterozygosity (LOH) in several cancer types and believed to harbor tumor suppressor genes. Cystatin M was shown to directly inhibit the activity of cathepsins B, V, and L [18,19]. In addition, cystatin M controls the activity of legumain, which is a known oncogene and an indicator of poor prognosis in colorectal and breast cancer but was also found overexpressed in the majority of human solid tumors [20,21]. Thus, imbalance between proteases and their inhibitors cystatins can lead to tumor development, invasion and metastasis [22]. Analysis of the *CST6* gene shows a single CpG island with many potential methylation sites in the promoter and the exon 1 of the gene (~64 CpGs in a 507 bp segment) [23] and it was recently shown that this region is a target for DNA methylation, which results in loss of cystatin M expression in breast cancer lines and breast carcinomas [23-25].

We have previously demonstrated that *CST6* is hypermethylated in breast cancer tissues and that *CST6* promoter methylation provides important prognostic information in patients with operable breast cancer [26]. Moreover we have recently shown that *CST6* is epigenetically silenced in Circulating Tumor Cells (CTC) isolated from peripheral blood of operable and metastatic breast cancer patients [27]. Herein, we report a novel closed-tube MS-HRMA assay for the semi-quantitative determination of *CST6* promoter methylation in clinical samples. Moreover, performance of the developed *CST6* MS-HRMA assay is compared to the performance of our previously described methylation specific PCR for *CST6.*

## Methods

### Patients and samples

Our study material consisted of a total of 116 clinical samples: a) one pilot testing group, consisting of 36 samples: 10 paired breast cancer and 10 adjacent histologically normal non-cancerous tissues, 7 histologically cancer-free specimens obtained from healthy women during reduction mammoplasty, and 9 breast fibroadenomas (included as a separate benign tumor group) and b) one independent cohort consisting of 80 formalin fixed paraffin-embedded (FFPE) breast carcinomas, obtained from patients with operable breast cancer from the Department of Medical Oncology, University Hospital of Heraklion Crete. All samples were collected at diagnosis and all patients gave their informed consent to participate in the study which has been approved by the Ethical and Scientific Committees of our Institution. Tissue sections of 10 μm containing >80% of tumor cells were used for DNA extraction and for MS-HRM analysis. Genomic DNA (gDNA) from paraffin tissues was isolated with the High Pure PCR Template Preparation kit (Roche, Germany). DNA concentration was determined in the Nanodrop ND-1000 spectrophotometer (Nanodrop Technologies, USA). Before proceeding to the sodium bisulfite conversion and MSP reaction steps, the genomic DNA integrity of all our clinical samples was assessed by amplifying BRCA1 exon 20 for mutation analysis by using the same primers as previously described [28].

### Sodium bisulfite conversion

1 μg of extracted DNA was modified with sodium bisulfite (SB), in order to convert all unmethylated, but not methylated-cytosines to uracil. Bisulfite conversion was carried out using the EZ DNA Methylation Gold Kit (ZYMO Research Co., Orange, CA), according to the manufacturer’s instructions. The converted DNA was stored at −70°C until used. In each sodium bisulfite conversion reaction, dH_2_O and breast cancer cell line MCF-7 were included as a negative and positive control, respectively.

### Controls

Human placental genomic DNA (gDNA; Sigma-Aldrich) and Universal Methylated Human DNA Standard (ZYMO Research Co., Orange, CA), were used as fully unmethylated and fully methylated controls respectively. Both controls underwent sodium bisulfite conversion, and a series of synthetic controls containing 0%, 1%, 10%, 50% and 100% methylated DNA were prepared by spiking the fully methylated DNA control into the unmethylated. These synthetic methylated DNA controls were used for the evaluation of the sensitivity of the assay and the semi-quantitative estimation of *CST6* methylation in our clinical samples.

### Methylation sensitive high resolution melting (MS-HRM)

#### *In silico* primer design

**Figure 1:**
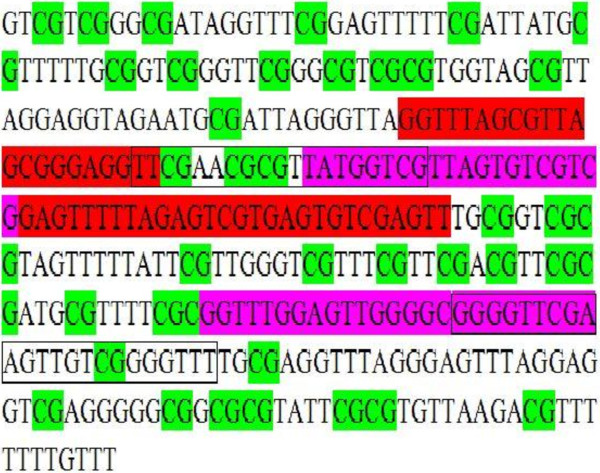
**The primers of MS-HRM and MSP assays for *CST6 *promoter methylation.** The MS-HRM primers are shown in red. The outer MSP primers are framed, while the inner ones are shown in purple. The region from −162 up to +188 is depicted (+1 is the Transcriptional start site). Note that this sequence is produced after bisulfite conversion of genomic DNA. All CpGs are considered to be methylated, and therefore are unaffected during the conversion process.

The primer set was designed *in silico,* using the PrimerPremier 5 software (Premier Biosoft International, USA), and synthesized by FORTH (Heraklion, Greece). During PCR the methylated and unmethylated templates have to be amplified equally so as the percentage of the methylated products reflects the percentage in the original sample. In low annealing temperatures bias favor the unmethylated template [29]. Therefore, the annealing temperature is critical. In order to reverse those PCR bias, improve the sensitivity of the assay and ensure that only SB converted DNA is amplified the primer set was designed according to the guidelines of Wojdacz et al. [6,30-33]. The sequence for the forward primer is 5′-GGTTTAGCGTTAGCGGGAGGTT-3′ and for the reverse primer is 5′-AACTCGACACTCACGACTCTAAAAACTC-3′. The PCR amplicon consists of 79 bp, (+9 up to +87; +1 being the transcriptional start site of *CST6* gene) [34]. The reverse primers are within the same region that was used for the nested MSP in the same samples as previously described [26]. The exact position of CGs in the *CST6* gene and the MS-HRMA and MSP primers used in this study are shown in Figure 1.

### PCR

Real-time PCR was performed in the LightCycler® 480 instrument (Roche Applied Science, Germany) using 96-well plates (Roche Applied Science, Germany). Extensive optimization experiments were performed in order to maximize PCR amplification efficiency, including PCR program parameters, Mg^2+^, primer and template concentrations. In addition optimization for the annealing temperature in order to reverse PCR bias as described above was carried out. 1 μL (~100 ng) of SB converted DNA was added in the PCR reaction mix, which consisted of 1X PCR Buffer (Invitrogen, USA), 0.4 mM for each dNTP (Invitrogen, USA), 0.05 U/μL Platinum® *Taq* DNA Polymerase (Invitrogen, USA), 0.25 μg/μL BSA (Sigma, Germany), 1X LCGreen Plus Dye (Idaho Technology, USA), 0.25 μM primers, and Mg^2+^ (2.5 mM). dH_2_O was used to supplement up to 10μL. The real-time PCR protocol began with one cycle at 95°C for 5 min followed by 50 cycles of: 95°C for 15 s, 60°C for 10 s and 72°C for 20 s. Immediately after amplification, a re-annealing cycle consisting of 95°C for 1 min and a rapid cooling to 70°C for 1 min was introduced in order to prepare the melting curve acquisition step. Real-time fluorescence acquisition was set at the elongation step (72°C). Samples whose amplification begun late or the relative fluorescence value on the raw melting-curve plot was low were not further processed. All PCR reactions were performed in triplicate for each sample.

### High resolution melting analysis

All assay optimization studies were performed first in the HR-1 High Resolution Melter (Idaho Technology, USA). For this reason, Real-time PCR was performed in the LightCycler 2.0 instrument using glass capillary tubes that were transferred after PCR to the HR-1 High Resolution Melter. Melting data acquisition began at 69°C and ended in 95°C, using a ramp rate of 0.3°C/s. High Resolution Melting Analysis was also performed in the LightCycler® 480 instrument (Roche Applied Science, Germany) using 96-well plates (Roche Applied Science, Germany). Data processing included normalization, and resulted on the normalized melting curves and the respective negative derivative of fluorescence over the temperature plots, using the LightCycler 480® gene scanning software. The settings for data collection were 50 fluorescence acquisition points per degree centigrade resulting on a ramp rate of 0.01°C/s. Comparison of the melting curve or the peaks of an unknown sample with those of the controls gave the semi-quantitative estimation for the methylation level of that sample.

## Results

### Assay optimization

Fully methylated and fully unmethylated DNA, as well as synthetic methylated DNA mixtures were used as controls for the optimization of the assay conditions, and evaluation of the analytical sensitivity and specificity of the MS-HRMA assay.

### Annealing temperature

Three different annealing temperatures were tested (60°C, 61°C, and 63°C). The normalized melting curves and the respective derivative plots, as obtained for the synthetic methylated DNA mixtures in all these three temperatures, were readily distinguishable from each other at 60°C ( Additional file 1 Figure S1).

#### Analytical sensitivity and specificity

**Figure 2:**
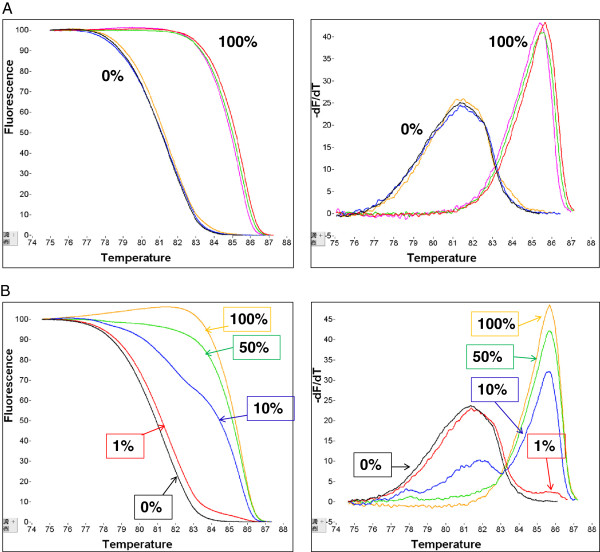
**Analytical validation of the MS-HRMA assay for *CST6 *promoter methylation: A) Specificity and reproducibility of the assay: 0%: human placental genomic DNA, 100% methylated control: universal methylated human DNA standard, run three times at three different days,B) Sensitivity, Black: 0%, red: 1%, blue: 10%, green: 50%, yellow: 100% methylation.**

The developed MS-HRMA assay for *CST6* methylation is highly specific for SB treated DNA since under these experimental conditions only SB treated DNA is amplified. When genomic DNA isolated from the A13 cell line that was not SB modified was added, amplification under the same conditions was not observed ( Additional file 2: Figure S2). We could readily discriminate between SB treated methylated and SB treated unmethylated controls and no dimers or “non-specific” products were observed. As can be seen in Figure 2A the unmethylated and the fully methylated SB treated DNA controls gave only one peak at their expected Tm values respectively. To evaluate the analytical sensitivity of the assay, dilutions of fully methylated to fully unmethylated DNA (1% - 100%) were assessed. The synthetic mixtures appeared having both peaks as expected. Fluorescence difference plots were generated and the ability to discriminate melting transitions of methylated DNA samples from that of unmethylated DNA samples was assessed. As can be seen in Figure 2B the presence of 1% of methylated *CST6* sequence can be easily verified in the presence of 99% unmethylated *CST6* sequence. When the analysis for the same control samples was repeated three times in three different days, melting curves were highly reproducible (Figure 2A).

Before applying the developed methodology in a high-throughput format, we compared our results for the same control samples using both a 96-well plate format LightCycler 480 (II) instrument (Roche, Germany), and the HR-1 instrument. Melting transitions presented almost identical profiles for both instruments (data not shown).

### Pilot testing group

In the pilot testing group, we analyzed by MS-HRMA for *CST6* methylation 10 paired breast cancer and 10 adjacent non-cancerous (histologically normal) tissues, 7 histologically cancer-free specimens obtained from healthy women during reduction mammoplasty, and 9 breast fibroadenomas (included as a separate benign tumor group). The methylation levels ranged from slightly lower than 1% up to approximately 50%. It is interesting to note that in the 10 paired breast cancer and 10 adjacent non-cancerous (histologically normal) tissues studied, in all cases where the tumor sample was found negative for methylation, the adjacent non-cancerous tissue was also negative ( Additional file 3: Table S1). In two cases, where the tumor samples were methylated at low percentage the adjacent non-cancerous tissue were also negative. Among the 10 adjacent to tumors non-cancerous (histologically normal) tissues tested only one was found to be methylated. It must be noted that especially in this case, the corresponding tumor sample was heavily methylated (approximately 50%), and the respective adjacent to the tumor sample showed only 1% methylation. None of the 7 (0%) histologically cancer-free specimens from reduction mammoplasty was found to be methylated for *CST6* promoter. However, one out of 9 fibroadenomas (11.1%) showed approximately 10% methylation for *CST6* promoter. Moreover, there was a very good concordance between MS-HRMA and MSP, since in 18/20 (90%) of these samples MS-HRMA gave the same results as MSP. There were only 2 samples, where MS-HRMA gave negative results while MSP was positive.

### Independent group

**Figure 3:**
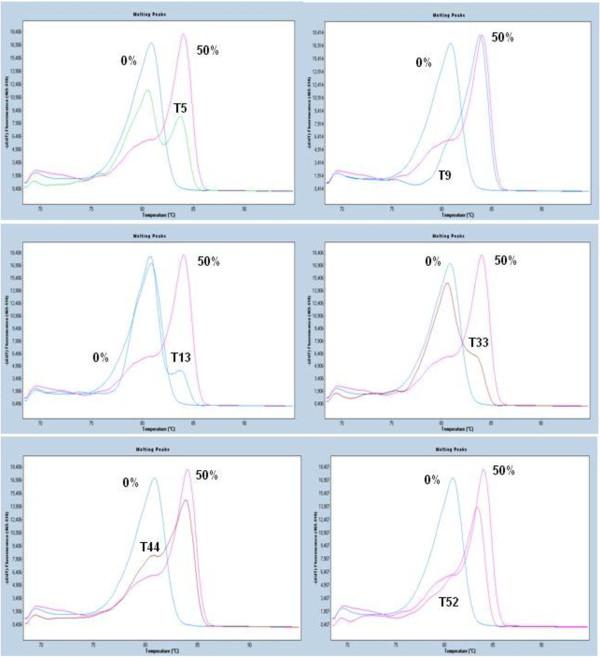
**Characteristic first derivative MS-HRMA plots for the semi-quantitative estimation of% methylation for *CST6 *promoter methylation by MS-HRMA in six tumor FFPE samples:T5:10%, T9: 50%, T13: 1%, T33: 5%, T44: 20% and T52: 20%.** Blue: 0%, Red: 50%.

We further applied the developed MS-HRMA assay to evaluate the *CST6* methylation status in an independent cohort consisting of 80 FFPE breast carcinomas samples. 39 out of the 80 tumor samples (48.75%) were found to be methylated. As can be seen in Figure 3, the melting patterns of the samples when compared to that of the spiked control samples with known percentages of *CST6* methylation, always run in parallel, allowed for their classification as non methylated or methylated, while the percentage of methylation could also be determined for the latter ones. The clinicopathological characteristics in respect to the methylation status of *CST6* of these patients are shown in Table 1. As can be seen in Table 1 there was no correlation between *CST6* methylation status and any clinicopathological parameter studied.

**Table 1:** **Association of *CST6 *methylation status with clinicopathological features for the independent group (n = 80)**

**Clinicopathological features**		***CST6*methylation**			
		**n**	**+ (%)**	**- (%)**	P value*
Age (years)	<55	42	22 (52.4)	20 (47.6)	0.495
	≥55	38	17 (44.7)	21 (55.3)	
Menopausal status	Pre	34	18 (52.9)	16 (47.1)	0.519
	Post	46	21 (45.7)	25 (54.3)	
Tumor size (cm)	0-2.0	23	11 (47.8)	12 (52.2)	0.682
	2.1-5.0	48	22 (45.8)	26 (54.2)	
	>5.0	8	5 (62.5)	3 (37.5)	
Axillary lymph node	0	23	9 (39.1)	14 (60.9)	0.366
	1-3	28	14 (50.0)	14 (50.0)	
	≥4	27	16 (59.3)	11 (40.7)	
Tumor grade	Ι	22	8 (36.4)	14 (63.6)	0.172
	ΙΙ, ΙΙΙ	58	31 (53.4)	27 (46.6)	
Estrogen receptor	Positive	49	22 (44.9)	27 (55.1)	0.386
	Negative	31	17 (54.8)	14 (45.2)	
Progesterone receptor	Positive	26	10 (38.5)	16 (61.5)	0.201
	Negative	54	29 (53.7)	25 (46.3)	

*: Chi-square test.

**Figure 4:**
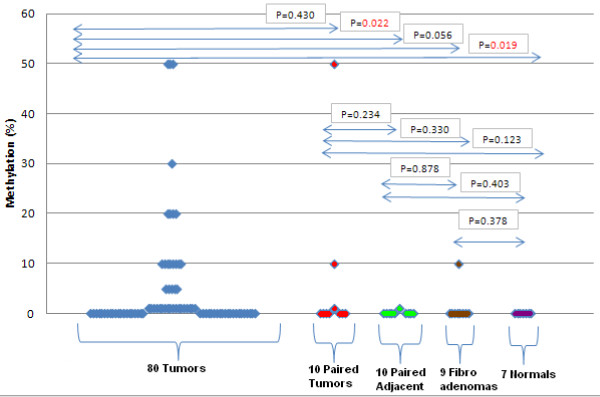
***CTS6 *methylation levels as estimated by MS-HRMA in the pilot testing group and independent group.** P values estimated by the Mann–Whitney test.

Finally, a graph presenting the methylation percentage of each sample across various sample categories, is shown in Figure 4. Mann–Whitney test was performed to evaluate whether a significant difference in methylation levels between those groups exist. As can be seen in this figure, the methylation levels for these 80 tumor FFPE samples were significantly different than those of the 10 non-cancerous adjacent to tumor tissues, and the 7 non-cancerous samples, belonging to healthy persons that underwent mammoplasty surgery, while there was not a significant difference between these samples and the 10 tumors of the independent group as well as with the 9 fibroadenomas tested, since one of them was highly methylated (10%). Nevertheless, the small number of available fibroadenomas and normal samples do not allow us to have a clear view in respect to those two categories.

### Comparison between MS-HRMA assay and MSP

In the pilot testing group, when all samples were also analyzed by our previously reported MSP assay [26] we found comparable results between the two assays. More specifically, 29 samples were found negative and 5 samples were found positive by both assays, while only 2 samples were positive for MSP and negative for MS-HRMA and no sample was positive by MS-HRMA and negative by MSP. In the independent group, when all these samples were also analyzed by our previously reported MSP assay [26] we also found comparable results between the two assays. More specifically, 21 samples were found negative and 29 samples were found positive by both assays, while 20 samples were positive for MSP and negative for MS-HRMA and 10 samples were positive by MS-HRMA and negative by MSP. In total, for 84/116 (72.4%) samples the two methods gave comparable results, (Table 2). More specifically, 50 samples were found negative and 34 samples were found positive by both assays, while 22 samples were positive for MSP and negative for MS-HRMA and 10 samples were positive by MS-HRMA and negative by MSP. For comparison of these two methods we used the Mac Nemar test which is a non-parametric method used on nominal data. According to this test the null hypothesis of marginal homogeneity states that the two marginal propabilities for each method are the same. The resulting P value using a binomial distribution, (P = 0.050) indicated that the two methods are giving comparable results. Moreover, we have evaluated the agreement between these two methods by calculating the kappa index adjusted for a 2-way comparison. This index that has been developed as a measure of agreement that is corrected for chance and according to the Guidelines for Strength of Agreement Indicated with Κ Values, the resulting kappa value of 0.4436 is indicative of a moderate agreement between these two methods [35]. Kappa index was calculated according to a program that is available online (http://vassarstats.net/kappa.html) while statistical analysis was performed using the SPSS Windows version 17.0 (SPSS Inc., Chicago, IL).

**Table 2:** Contingency table which tabulates the outcomes of both methods for all samples tested and kappa index values (n = 116)

**Method**		**MSP**		**Total**
		**pos**	**neg**	
MS-HRM	pos	34	10	44
	neg	22	50	72
total		56	60	116
**Indices of agreement for MS-HRMA and MSP for*CST6*methylation**				
**Agreement index**	**Type of agreement**	**Calculated values**	**Standard error**	**CI (95%)**
p_o_	overall	0.7241		
p_pos_	positive	0.6800		
p_neg_	negative	0.7575		
p_e_	chance	0.5041		
Kappa index	Chance corrected	0.4436	0.0817	0.2834 – 0.6038

P =0.050, (Mac Nemar test , binomial distribution used.).

## Discussion

Cystatin M, originally described as a putative tumor suppressor, whose expression is often diminished or completely lost in metastatic breast cancers [15] has been clearly shown to be epigenetically regulated by strong hypermethylation of the *CST6* gene promoter in breast cancer cell lines [23], in breast cancer and metastatic lesions in the lymph nodes [34], in malignant gliomas [36], in cervical [37] and prostate cancer [38]. Because promoter hypermethylation does not account for the loss of *CST6* expression in all tumors alternative modes of *CST6* repression are likely, such as histone deacetylation and repressive chromatin structure may be involved [37], since silencing of *CST6* has been associated with repressive trimethyl-H3K27 and dimethyl-H3K9 histone marks [39].

Recently, *CST6* was also identified among 10 hypermethylated genes that distinguish between cancerous and normal tissues according to the extent of methylation [40]. Moreover, a whole-genome approach using a human gene promoter tiling microarray platform to identify genome-wide and gene-specific epigenetic signatures of breast cancer metastasis to lymph nodes led to functional associations between the methylation status and expression of genes *CDH1, CST6, EGFR, SNAI2 and ZEB2* associated with epithelial-mesenchymal transition [41]. In addition, a recent functional epigenetic study of renal cell carcinoma (RCC) cell lines and primary tumors by high-density gene expression microarrays identified *CST6* as one of eight genes that showed frequent (>30%) tumor-specific promoter region hypermethylation associated with transcriptional silencing (epigenetically inactivated candidate RCC TSGs). According to this study, re-expression of *BNC1, CST6, RPRM and SFRP1* suppressed the growth of RCC cell lines [42]. All these recent studies are in support of the importance of *CST6* promoter methylation in metastasis.

Our group has shown for the first time the prognostic significance of *CST6* promoter methylation in patients with operable breast cancer [26]. According to our findings, the diagnostic sensitivity and specificity of *CST6* methylation as a biomarker for prediction of relapses and deaths in operable breast cancer seems to be quite promising [26]. Moreover, we have recently shown that *CST6* promoter was methylated in Circulating Tumor Cells (CTC) isolated from peripheral blood of breast cancer patients, in both groups of early disease and verified metastasis [27]. A recent study has also shown that cystatin M loss may be associated with the losses of ER, PR, and HER4 in invasive breast cancer [43].

Based on all these studies, we strongly believe that the reliable and easy detection of *CST6* methylation in clinical samples will be of great importance for cancer research. For this reason we decided to develop a closed tube, highly sensitive, cost-effective, rapid and easy-to-perform assay for *CST6* promoter methylation based on methylation-sensitive high resolution melting analysis (MS-HRMA). Resolution of DNA methylation by melting analysis relies on the fact that the *T*m of a PCR product generated from bisulfite-treated DNA reflects the methylation status of the original DNA template [44]. Because unmethylated cytosines will be converted into uracil during bisulfite treatment and subsequently amplified as thymine, whereas methylcytosines will remain as methylcytosine and be amplified as cytosine, the methylated sequence will have a higher G:C content, and hence a higher Tm, than the corresponding unmethylated sequence. After amplification with primers that will not differentiate between methylated and unmethylated molecules, the melting properties of the PCR products can be examined in the thermal cycler by slowly elevating the temperature under continuous or step-wise fluorescence acquisition. The melting curves or derived melting peaks provide a profile of the methylation status of the entire pool of DNA molecules in the sample [44].

Many reports have already clearly illustrated the great potential of melting analysis for sensitive and highthroughput assessment of DNA methylation in inherited disorders and cancer [6,11-13,30-33,44]. Compared with current gel-based assays MS-HRMA has the important advantage of the closed-tube format, which simplifies the procedure, decreases the risk of PCR contamination, and decreases analysis time. In addition, melting analysis resolves heterogeneous methylation, detects methylated and unmethylated alleles in the same reaction, and requires only standard, inexpensive PCR reagents. In addition, the design of individual assays is simple [45-47].

The developed assay is highly specific and sensitive since it can detect the presence of low abundance *CST6* methylated DNA sequences (down to 1%). Moreover to the best of our knowledge, this is the first assay reported so far that provides additionally a semi-quantitative estimation of *CST6* promoter methylation. When compared to MSP, the developed MS-HRMA gives comparable but not identical results. The discrepancies between MS-HRMA and MSP can be explained by the different principles on which these methods are based. In MSP we get a positive signal only when the specific CpG island that the primers are designed for is methylated. However it is known that different samples can vary in the methylation sites in specific positions in their CpG islands. In this way if a sample is methylated in positions 3, 6 and 7 and the MSP primers are designed to recognize methylation in positions 4, 5 and 8, MSP will give a negative result, while MS-HRMA will give a positive result since it is affected by the presence of any methylated CpG island that is located between the primers. In the opposite way, if the methylation sites that are recognized by the MSP primers are not included in the region amplified by MS-HRMA primers a sample found positive by MSP will be negative by MS-HRMA.

This is the first time that methylation levels for *CST6* are reported in clinical samples. Based on our findings, we can definitely say that these levels vary significantly among samples. An interesting finding is that a histologically “non-cancerous” tissue that was adjacent to a highly methylated (50%) tumor sample was also found to be methylated, at a lower percentage (1%). *CST6* methylation is an early event in breast cancer, since methylation of the *CST6* promoter has already been reported in 7 out of 28 corresponding normal tumor-adjacent breast tissues samples [25]. This could possibly indicate that some “normal” cells surrounding the tumor tissue have already a malignant transformation, not detected by conventional immunohistochemistry. In our study we have used whole tissue sections containing more than 80% of tumour cells. However, we can speculate that the percentage of contaminating normal cells affect the level of methylation seen in our samples. For this reason, we believe that laser capture microdissection could ensure a higher proportion of lesional cells in clinical samples to be studied.

## Conclusions

The developed methylation-sensitive high resolution melting assay (MS-HRMA) for the semi-quantitative determination of *CST6* promoter methylation can be a very useful tool to evaluate reliably and semi-quantitatively *CST6* methylation in a variety of clinical samples. Moreover it is a closed tube assay, easily applicable in many real time PCR instruments equipped with high resolution melting analysis software, cost-effective, rapid and easy-to-perform. It gives comparable results to MSP in less time, while it offers the advantage of additionally providing an estimation of the level of methylation.

## Abbreviations

MSP: Methylation-specific PCR; MS-HRMA: Methylation-sensitive high-resolution melting analysis; CST6: Cystatin M gene; SB: Sodium bisulfite; MIP: methylation independent PCR; HRMA: High-resolution melting analysis; FFPE: Formalin fixed paraffin-embedded; CTC: Circulating tumor cell.

## Competing interests

The authors declare that they have no competing interests.

## Authors’ contributions

LD and PV have made substantial contributions to the analysis and acquisition of data, VG has provided the clinical samples and has been involved in drafting the manuscript and EL conceived of the study, and participated in its design and coordination and helped to draft the manuscript and has given the final approval of the version to be published. All authors read and approved the final manuscript.

## Pre-publication history

The pre-publication history for this paper can be accessed here:

http://www.biomedcentral.com/1471-2407/12/486/prepub

## Supplementary Material

Additional file 1 ** Figure S1. **Optimization of the annealing temperature of the MS-HRMA assay for *CST6* promoter methylation. Normalized melting curves and first derivative plots for a) 63ºC: Black: 0%, red: 1%, blue: 10%, green: 50%, yellow: 100% methylation b) 61ºC: Black: 0%, red: 1%, blue: 10%, yellow: 50%, green: 100% methylation and c) 60ºC: Black: 0%, red: 1%, blue: 10%, green: 50%, yellow: 100% methylation. Click here for file

Additional file 2 ** Figure S2. **Specificity of MS-HRMA assay for *CST6* promoter methylation: PCR products of the SB modified positive controls and genomic DNA (unconverted). 1) DNA ladder 2) negative control (H_2_O), 3) 0% methylated control 4) 1% methylated control 5) 10% methylated control 6) 50% methylated control 7) 100% methylated control 8) genomic DNA (unconverted). Click here for file

Additional file 3 ** Table S1. ***CST6* methylation status in 10 paired breast cancer and 10 adjacent non-cancerous tissues as evaluated by both the developed MS-HRMA and MSP [26] assays. Click here for file
