# Supplementary figures and images for: A closed-tube methylation-sensitive high resolution melting assay (MS-HRMA) for the semi-quantitative determination of CST6 promoter methylation in clinical samples
Source: BMC Cancer. 2012 Oct 22;12:486. doi: 10.1186/1471-2407-12-486 (PMC3495665; doi:10.1186/1471-2407-12-486)

# Suppl.Figure 1

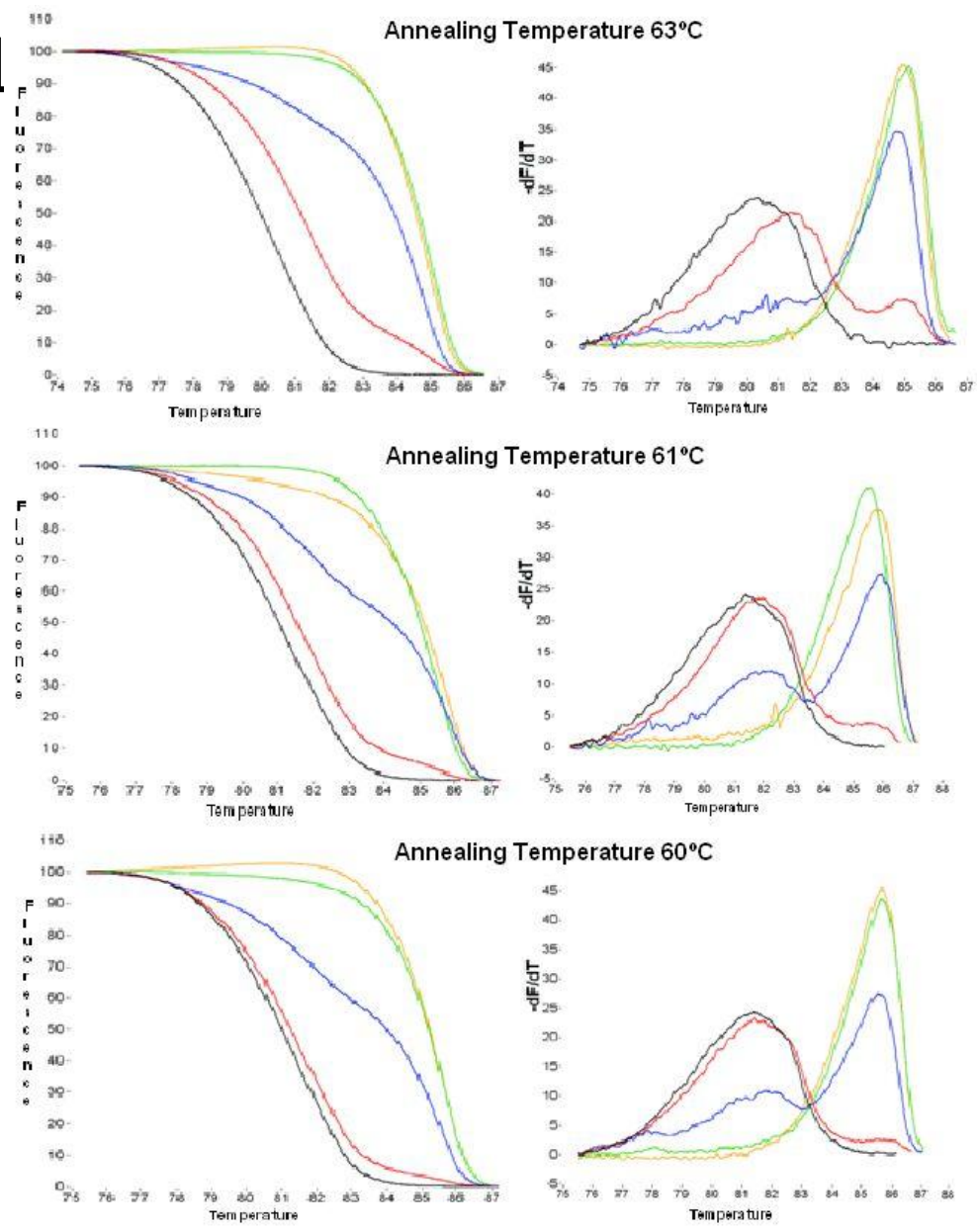

Supplement: Additional file 1 — Figure S1. Optimization of the annealing temperature of the MS-HRMA assay for CST6 promoter methylation. Normalized melting curves and first derivative plots for a) 63ºC: Black: 0%, red: 1%, blue: 10%, green: 50%, yellow: 100% methylation b) 61ºC: Black: 0%, red: 1%, blue: 10%, yellow: 50%, green: 100% methylation and c) 60ºC: Black: 0%, red: 1%, blue: 10%, green: 50%, yellow: 100% methylation. [file 1471-2407-12-486-S1.pdf]

# Suppl. Figure 2

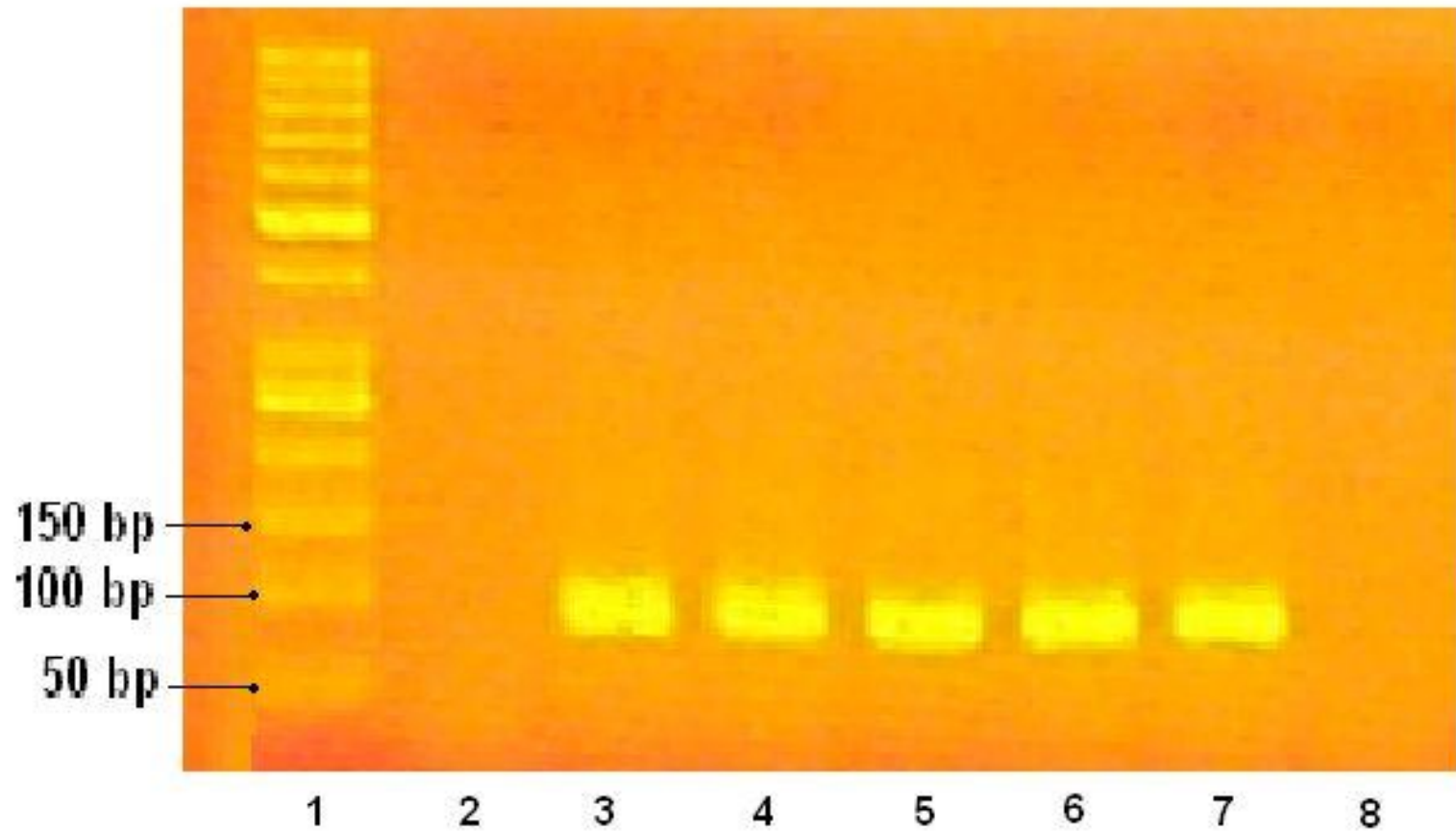

Supplement: Additional file 2 — Figure S2. Specificity of MS-HRMA assay for CST6 promoter methylation: PCR products of the SB modified positive controls and genomic DNA (unconverted). 1) DNA ladder 2) negative control (H2O), 3) 0% methylated control 4) 1% methylated control 5) 10% methylated control 6) 50% methylated control 7) 100% methylated control 8) genomic DNA (unconverted). [file 1471-2407-12-486-S2.pdf]
