# Supplementary material for: A closed-tube methylation-sensitive high resolution melting assay (MS-HRMA) for the semi-quantitative determination of CST6 promoter methylation in clinical samples
Source: BMC Cancer. 2012 Oct 22;12:486. doi: 10.1186/1471-2407-12-486 (PMC3495665; doi:10.1186/1471-2407-12-486)
Supplement: Additional file 3 — Table S1. CST6 methylation status in 10 paired breast cancer and 10 adjacent non-cancerous tissues as evaluated by both the developed MS-HRMA and MSP [26] assays. [file 1471-2407-12-486-S3.docx]

**Suppl. Table 1.** *CST6* methylation status in 10 paired breast cancer and 10 adjacent non-cancerous tissues as evaluated by both the developed MS-HRMA and MSP (26) assays.

| Sample Code | Tumor Samples | | | Adjacent non-cancerous tissues | | |
| --- | --- | --- | --- | --- | --- | --- |
| **Paired tumor tissues** | MSP | MS-HRMA | Methylation % (HR) | MSP | MS-HRMA | Methylation % (HR) |
| 1 | + | + | 1% | - | - | 0% |
| 2 | - | - | 0% | - | - | 0% |
| 3 | + | + | 10% | - | - | 0% |
| 4 | + | + | 50% | + | + | 1% |
| 5 | - | - | 0% | - | - | 0% |
| 6 | - | - | 0% | - | - | 0% |
| 7 | - | - | 0% | - | - | 0% |
| 8 | - | - | 0% | - | - | 0% |
| 9 | + | - | 0% | - | - | 0% |
| 10 | + | - | 0% | - | - | 0% |
